# Issues in patient identification during COVID-19

**DOI:** 10.1097/01.NURSE.0000820068.71332.77

**Published:** 2022-02-24

**Authors:** Tracy Jones-Darnell

**Affiliations:** **Tracy Jones-Darnell** is a contributing faculty at Walden University in Minneapolis, Minn.

**Keywords:** COVID-19, dementia, long-term-care facility, medical error, patient identification, patient misidentification

## Abstract

This article presents cases of patient misidentification during the onset of the COVID-19 pandemic to illustrate the critical importance of positive patient identification.

**Figure:**
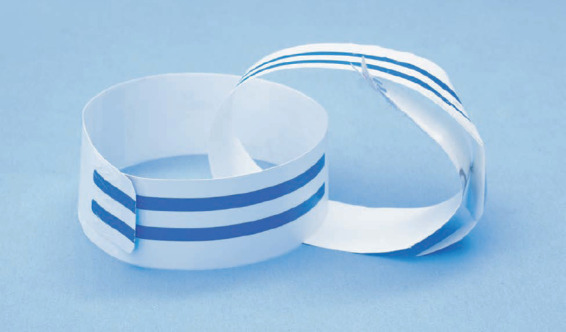
No caption available.

Long-term-care (LTC) facilities were one of the most impacted by the COVID-19 pandemic. By mid-April 2020, over 7,000 patients and staff had died from the virus and another 36,500 had been infected with COVID-19 in 4,100 LTC facilities across the United States.^1^ These unprecedented times brought unimaginable suffering for patients, staff, and their families. The sudden and drastic high infection rate in these facilities was partly because patients are in constant, daily contact with the staff and other patients.^2^ Some would equate this environment as familial as the patients and staff have been together for years.

LTC facilities reported to have initiated crisis care without a collection of trusted resources and best practices specific to LTC settings.^1^ Decisions were made using a variety of sources such as the CDC, The Society for Post-Acute and Long-Term Care Medicine, the Department of Health and Human Services, and the National Academy of Medicine (formerly called the Institute of Medicine).^1^ Facilities struggled with inadequate staffing and isolation implementation in communal living environments.

Patients in LTC are medically vulnerable due to their advanced age and underlying comorbidities. Patients with dementia are some of the most vulnerable patients. As of January 2022, the highest number of COVID-19 deaths in the US was among individuals 85 years of age or older.^3^ Some of the first cases of COVID-19 in the US were diagnosed in an LTC facility. This article presents cases of patient misidentification during the onset of the COVID-19 pandemic to illustrate the critical importance of positive patient identification.

## Cases of patient misidentification

CL and TH were residents in a large, multifloor LTC facility in New York state during the COVID-19 pandemic. Both patients were White males in their early 80s with moderate dementia. Both men had supportive families who often visited them prior to the strictly enforced no-visitor policy. The resulting scenario is an egregious example of the breakdown in following the core tenets of patient identification that are taught in nursing school.

In late March 2020, during the height of the COVID-19 pandemic in New York State, CL and TH began to exhibit respiratory signs and symptoms of COVID-19. As a precautionary measure, the facility began quarantining patients who displayed signs and symptoms of COVID-19 to a specific unit while awaiting their polymerase chain reaction (PCR) test results. Prior to their respiratory issues, CL and TH were in good physical condition and received supervised care due to their moderate dementia.

On a Tuesday morning in March, CL and TH were transferred to a designated COVID-19 unit. This unit was closed off from the rest of the facility and had a different group of staff assigned to care for its resident. CL and TH received new ID bands including their new unit and room numbers.

It was during this step in the transfer process that it is believed these patients were given the wrong ID bands. It is important to note that while patient wrist bands are not patient identifiers, they are often scanned or used prior to providing nursing care or administering medications. Several factors could be attributed to the misidentification amid the chaos that had overtaken the facility.

The patients' families were barred from entering the facility in an effort to avoid infection. The families were instructed to call daily for status updates on each patient's condition.

CL's son said he called every day to get updates on his father, but he complained about a near-blackout of communications from the facility. He reported the phones would go unanswered and his voicemails were not returned. He stated that when someone did answer his call, it was often a hurried conversation during which little or confusing information was conveyed.

Unfortunately, after his condition declined rapidly from COVID-19, TH passed away just 3 days after being moved to the COVID-19 unit. TH's family was notified of his death and instructed to notify the funeral home director that the body could be claimed after the mandatory decontamination period was over. His family mourned for his death and made the necessary arrangements.

Prior to the pandemic, the deceased person's family would go into the facility to say their last goodbyes before the funeral home picked up the body, but due to the COVID-19 restrictions, the family had to wait to see the body at the funeral home. The funeral home was later notified when the body was ready to be released. This was the point when everything changed, forever altering the lives of many due to misidentification.

Although TH had passed away from COVID-19, CL's health was improving and when his son called for a progress update, he was given the good news.

It is important to note that the two patients had vastly different final wishes for their bodies. CL's body, which everyone thought was TH's body at that time, was taken to the funeral home to be prepared for cremation, per TH's final wishes. However, CL had instructed his son that his final wishes were to be buried next to his wife in their hometown.

The employee who was preparing the body at the funeral home noticed two medical bracelets: one on the wrist and one on the upper arm near the elbow, each with a different name. The employee notified the funeral director, who then called the LTC facility to verify the body's identity before proceeding.

The facility verified that misidentification had occurred. Unfortunately, there had been a mix-up. In reality, it was actually CL who had passed away and TH whose health was improving. The facility's manager called CL's son and told him there had been a mistake, and despite having been told his condition was improving, his father had actually passed away the previous week. After the initial shock, CL's son requested that the funeral home director email him photos of the deceased person to verify his identity. Unfortunately, he confirmed from the pictures that the body was, in fact, his father.

The shaken funeral home director summed up the situation: “You have one family who was told that their loved one was dead when he wasn't, and you have another family who was told their loved one was alive when he wasn't. I've never seen anything like this.”

“They gave us hope and it was total misinformation,” shared CL's son. “This was crushing to my family.”

## Recommendations

In LTC facilities, it is common practice to have a picture of a patient with dementia in their medical record as an additional layer of patient identification. In the case of CL and TH, the patients had similar physical characteristics and health histories and were wearing face masks, which obscured their faces and may have perpetuated the misidentification. The facility's no-visitor policy—with patients' families unable to see their loved ones—may have contributed to the difficulty as well.

The breakdown of the patient identification procedure may have occurred in various areas. For example, the patients' pictures may not have been verified. Had appropriate procedures been followed, the misidentification would have been caught in the early stages before CL and TH were treated erroneously for days, receiving the other's medications and treatments.

As with many healthcare errors, this misidentification case significantly impacted the patients' families and the staff involved. Using this case as an example, healthcare workers can urge national leaders to enact policy measures to ensure adequate staffing and education for healthcare workers caring for vulnerable patient populations.

In the fall of 2020, the US Department of Health and Human Services recognized the negative impact of COVID-19 on patient safety in LTC facilities, and, with the help of the Agency for Healthcare Research and Quality, created the National Nursing Home COVID-19 Action Network.^5^ This network provides free staff training to assist LTC facilities with implementing evidence-based patient safety practices to protect patients. This training also boosts the staff's competence in caring for patients with dementia.

Prevailing factors that have been reported to lead to patient misidentification include higher than normal patient loads, communication gaps, and reliance on patients to confirm their identity, as evidenced in this case.^5^ Misidentification most frequently occurs during the intake process. Most errors do not occur because providers are lax or because they adopt unsafe habits. Rather, they occur as a result of multiple factors, including systemic and organizational failures. Simply accusing or punishing direct providers does not prevent the next error.^5^ Patient safety cannot rely on human perfection in ordinary circumstances, much less during a crisis. A full root cause analysis is required to identify all contributing factors and create procedural safeguards that allow people to make inevitable human mistakes without harm reaching the patient.

First, the healthcare system should be evaluated for problems and gaps in nurse training. Nurses should never identify a patient by the room or bed number, diagnosis, or patient demographic such as “older White male with dementia.” Nurses should never ask, “Is your name...?” Nurses should not assume identity has already been confirmed by someone else or deviate from the patient identification policy of using two acceptable patient identifiers. Facilities also need to employ regular auditing to ensure that their staff is adhering to their patient identification practices.

In 2021, The Joint Commission published the Nursing Care Center National Patient Safety Goals to ensure adherence to correct patient identification protocols in LTC facilities.^6^ The Joint Commission and the World Health Organization recommend using at least two appropriate identifiers such as the patient's name and date of birth.^6^ Patient verification should be conducted even if the patient is familiar to healthcare professionals. When room changes are necessary, cohorting should be done within the same unit to keep the residents with familiar staff. The patient's room number or physical location is not an appropriate identifier, as this variable is fluid and can change from day to day and facility to facility.

Since the advent of digital charting, the use of bar code verification, although not available in all facilities, is an acceptable method of patient identification prior to the administration of care.^6^ The Joint Commission directs organizations providing care for individuals who are noncommunicative or have dementia to determine what process will be used to safely identify patients, clearly communicate these expectations to the staff, and ensure adherence. A photo of the patient is an example of a patient identifier that could have been used with the patients in this case study.

Lastly, the key recommendation for leaders in healthcare facilities is to communicate regularly with their staff regarding vigilance in patient identification.^7^,^8^ Leadership should seek input from staff about best practices and methods of incorporating this vital safety measure into their daily habits. Leadership should operate with an open-door policy and provide staff with support and assistance. Nurses have a duty to their patients to be accountable for nursing practices and ensure that patients receive the best and safest care possible
